# A fast monocular 6D pose estimation method for textureless objects based on perceptual hashing and template matching

**DOI:** 10.3389/frobt.2024.1424036

**Published:** 2025-01-08

**Authors:** Jose Moises Araya-Martinez, Vinicius Soares Matthiesen, Simon Bøgh, Jens Lambrecht, Rui Pimentel de Figueiredo

**Affiliations:** ^1^ Industry Grade Networks and Clouds, Institute of Telecommunication Systems, Electrical Engineering and Computer Science, Technical University Berlin, Berlin, Germany; ^2^ Future Manufacturing Technologies, Mercedes-Benz AG, Sindelfingen, Germany; ^3^ Department of Materials and Production, Aalborg University, Aalborg, Denmark

**Keywords:** 6D pose estimation, perceptual hashing, IoU, hamming distance, automotive production

## Abstract

Object pose estimation is essential for computer vision applications such as quality inspection, robotic bin picking, and warehouse logistics. However, this task often requires expensive equipment such as 3D cameras or Lidar sensors, as well as significant computational resources. Many state-of-the-art methods for 6D pose estimation depend on deep neural networks, which are computationally demanding and require GPUs for real-time performance. Moreover, they usually involve the collection and labeling of large training datasets, which is costly and time-consuming. In this study, we propose a template-based matching algorithm that utilizes a novel perceptual hashing method for binary images, enabling fast and robust pose estimation. This approach allows the automatic preselection of a subset of templates, significantly reducing inference time while maintaining similar accuracy. Our solution runs efficiently on multiple devices without GPU support, offering reduced runtime and high accuracy on cost-effective hardware. We benchmarked our proposed approach on a body-in-white automotive part and a widely used publicly available dataset. Our set of experiments on a synthetically generated dataset reveals a trade-off between accuracy and computation time superior to a previous work on the same automotive-production use case. Additionally, our algorithm efficiently utilizes all CPU cores and includes adjustable parameters for balancing computation time and accuracy, making it suitable for a wide range of applications where hardware cost and power efficiency are critical. For instance, with a rotation step of 10° in the template database, we achieve an average rotation error of 10° , matching the template quantization level, and an average translation error of 14% of the object’s size, with an average processing time of 0.3s per image on a small form-factor NVIDIA AGX Orin device. We also evaluate robustness under partial occlusions (up to 10% occlusion) and noisy inputs (signal-to-noise ratios [SNRs] up to 10 dB), with only minor losses in accuracy. Additionally, we compare our method to state-of-the-art deep learning models on a public dataset. Although our algorithm does not outperform them in absolute accuracy, it provides a more favorable trade-off between accuracy and processing time, which is especially relevant to applications using resource-constrained devices.

## 1 Introduction

The estimation of the six degrees-of-freedom (6DOF) pose of objects from image data is known as object 6D pose estimation and stands as a fundamental problem in various fields such as computer vision, robotics, augmented reality, and industrial automation.

The ability to precisely determine an object’s position and orientation in its environment plays a pivotal role in enabling machines to interact intelligently with the physical world. In robotics, robots equipped with vision systems rely on precise pose estimation to manipulate objects with dexterity and efficiency. Augmented reality applications leverage 6D pose estimation to seamlessly integrate virtual objects into real-world environments, enhancing user experiences. Industrial automation relies on accurate pose estimation for tasks such as assembly and quality control in production lines.

Despite its significance, the accurate estimation of the 6D pose is hindered by various challenges inherent to real-world scenarios, which include variability in lighting conditions, occlusions, cluttered backgrounds, changes in object appearance, and viewpoint alterations. These factors introduce ambiguity and complexity, making it difficult for algorithms to robustly infer the object’s pose solely from image data. For instance, an object’s appearance may change due to different lighting conditions or occlusions, and background clutter can confuse algorithms by introducing false positives. Although the literature on object detection and 6D pose estimation is vast (Marullo et al., 2022; Guan et al., 2024) and popular state-of-the-art methods are accurate when dealing with textured objects, most fail in the presence of textureless objects, such as metallic parts, which are common in industrial assembly and quality inspection lines (e.g., in the automotive industry).

In this work, we tackle the former problem by proposing a fast and robust template-matching methodology for textureless (metallic) 6D pose estimation, using a single conventional RGB camera. Similar to the work by Druskinis et al. (2023), we propose a model-based template matching approach for textureless object pose estimation, with benefits of perceptual hashing (Farid, 2021), to cleverly select only a subset of all possible templates for the template-matching routine. Unlike state-of-the-art data-driven deep learning methods, which are data and computationally hungry, our method only requires CAD models of the objects of interest for training, and our multi-threading implementation runs in real-time without the need for expensive and power-demanding GPUs.

Our main contributions, compared to our previous work (Druskinis et al., 2023), are as follows:

• We propose a method for generating a database of silhouettes, representing different 3D viewpoints, from a given object geometric (CAD) model. Viewpoints are deterministically sampled, instead of randomly, from all possible orientations.
• We propose the use of binary masks for representing the object instead of edges. Furthermore, we use the intersection over union for fast template matching instead of computationally expensive Chamfer distance between edges.
• We propose a novel perceptual hashing of binary image algorithms for preselecting a subset of the templates on the database for matching at run time. Our method shows improvements in several orders of magnitude in processing times compared to similar previous methods (Druskinis et al., 2023).
• A systematic set of experiments using simulated data demonstrates the robustness of our algorithm in the presence of occlusion and noise if optimized for speed.

The rest of this paper is organized as follows: Section 2 reviews the related work on computer vision methods for 6D pose estimation; Section 3 presents our perceptual hashing and template-matching approach for fast 6D object pose estimation; Section 4 presents the test of the robustness of our methods against noise and occlusion, as well as its run-time inference speed on different (workstation and embedded computing) devices; and finally, Section 5 presents the main benefits and setbacks of our approaches and proposes ideas for future work.

## 2 Related work

The literature on methods for 6D pose estimation of objects from color and depth image data is vast and spans across multiple fields (see Sahin et al., 2020; Zhao et al., 2019; Du et al., 2021; Marullo et al., 2022 for detailed reviews). Generally, these approaches can be split into three categories, namely, template-based, feature-based, and data-driven methods. Template-based methods create 2D object representations from many viewpoints, which are matched against a given input target image. However, these are typically prone to errors in the presence of occlusions and clutter. Feature-based methods detect and extract local features from the object. The 6D pose is typically retrieved using PnP algorithms. Unlike template-based methods, these are robust to occlusions and clutter but require rich textures to allow for features to be extracted. Data-driven methods learn to perform feature extraction and pose estimation from annotated datasets. Instead of engineering features, one utilizes neural network architectures (e.g., deep convolutional neural networks (Indolia et al., 2018)), which are optimized with large datasets to perform object detection and pose estimation in an end-to-end manner.

Zakharov et al. (2019) introduced a method called the Dense Pose Object Detector (DPOD), a deep learning method for 3D object detection and 6D pose estimation from RGB-D images. The DPOD utilizes dense multi-class 2D–3D correspondence maps to estimate object poses using PnP (Fischler and Bolles, 1981a) and RANSAC (Fischler and Bolles, 1981b) algorithms. Additionally, it uses a deep learning-based refinement scheme for further improving pose accuracy. Evaluation of both synthetic and real data shows superior performance compared to recent detectors, with real-time capability.

Choi and Christensen (2010) proposed a real-time 3D model-based tracking system designed for robotic manipulation tasks. The method leverages edge and key point features for robust tracking. The system uses a 3D model of the object to be tracked and utilizes edge and key point features extracted from the object and the scene. These features are matched in real time to estimate the object’s pose relative to the camera. This approach enables accurate and efficient tracking suitable for robotic manipulation applications.

Drost et al. (2010) and de Figueiredo et al. (2015) proposed a method for recognizing free-form 3D objects in point clouds. Unlike traditional approaches relying on local point descriptors, their method constructs a global model description using oriented point pair features and uses a fast-voting scheme for local matching. Recognition occurs locally via an efficient Hough voting scheme on a two-dimensional search space. The experimental results demonstrate high speed and high recognition performance in the presence of noise, clutter, and partial occlusions.

Although traditional methods for 6D pose estimation may achieve high accuracy, they are typically not robust to appearance variations and require a significant amount of engineering effort to design and optimize feature extractors, object proposals, and their classes (O’Mahony et al., 2020). More recent data-driven deep learning methods learn to perform complex visual recognition tasks, including 6D pose estimation, in an end-to-end manner by training neural network models with large, annotated datasets.

Pix2Pose (Park et al., 2019) is a deep learning method specifically engineered for 6D pose estimation from individual RGB images. Utilizing a convolutional neural network (CNN) architecture, Pix2Pose is trained end-to-end to directly predict the 6D pose parameters of objects depicted in the input images. During training, the network learns to minimize the disparity between its predicted poses and groundtruth annotations, typically comprising object translations and rotations relative to a reference frame. To enhance robustness and generalization, Pix2Pose incorporates data augmentation techniques, applying random transformations to both the input images and corresponding pose annotations.

Sundermeyer et al. (2019) proposed an approach for 6D object detection that utilizes augmented autoencoders to implicitly learn the 3D orientation of objects from 2D images. By augmenting autoencoders with orientation information during training, the proposed approach effectively encodes and decodes 3D object representations, facilitating the improved detection of object poses in 3D space. This is achieved by embedding orientation information directly into the latent space of the autoencoder, enabling the model to learn robust features for orientation estimation.

Wu et al. (2022) introduced pose interpreter networks (PINs) for efficiently predicting the 3D pose of objects from 2D images in real time. Unlike conventional methods that rely on computationally expensive geometric calculations or complex network architectures, PINs use a lightweight structure combined with a learnable pose interpreter module. This module refines pose predictions dynamically based on contextual information extracted from input images, ensuring accurate and robust pose estimation. The experimental results demonstrate that PINs outperform existing methods in terms of both accuracy and computational efficiency, making them suitable for applications such as robotics, augmented reality, and industrial automation.

Deep-IRTarget (Zhang et al., 2022) is a novel backbone network that detects targets using infrared images. The challenge of using CNNs in thermal imagery is due to poor texture information, low resolution, and high noise levels, which restrict the feature extraction ability of CNNs. Thus, Deep-IRTarget uses features from the frequency domain and spatial domain and stacks them together to construct dual-domain features. This approach offers a solution using infrared images and can be used as the object detection stage of a pose estimation algorithm to detect key features in objects without the need for RGB images.

MegaPose is a method introduced by Labbé et al. (2022) to estimate the 6D pose of novel objects using RGB or RGB-D images. Novel objects are those that are unseen during training, which eliminates the requirement of having to re-train the model for every new object that is added after training. MegaPose only assumes knowledge about a region of interest that contains the desired object in the image, along with its CAD model. To train the coarse and refiner models to generalize to novel objects, they required RGB-D images of many objects with their groundtruth 6D object pose annotations along with 3D models for these objects. To solve this task, they trained their models on purely synthetic data using BlenderProc (Denninger et al., 2023a), a procedural Blender pipeline for photorealistic rendering that provides 6D pose estimation data in the BOP format. Two million images were generated for their dataset by randomly sampling objects and dropping them from a plane using a physics simulator.

Labbé et al. (2020) introduced CosyPose, an approach to recover the 6D pose of multiple known objects captured by multiple input images, where the camera viewpoints are unknown and no depth information is given. It is assumed that the 3D models of the objects are known; however, there can be multiple instances of the object in the same, and the amount is unknown. Since multiple views of the scene are captured, some objects may not be visible in some scenes, and the relative poses between the cameras are unknown. The output of CosyPose is a scene containing the number of objects and their class along with their 6D pose and the relative poses of the cameras.

Hinterstoisser et al. (2013) created the Linemod dataset, which is a large dataset of 15 registered video sequences containing 15 textureless household objects with discriminative color, shape, and size. Every video sequence contains over 1,100 real images taken from multiple viewpoints, where one video sequence is associated with one of the 15 household objects and only has annotations for that object. Each sequence also uniformly covers views around the complete pose space of the object. This guarantees views from 0° to 360° around the object, 0°–90° tilt rotation, 65 cm–115-cm scaling, and ±45° in-plane rotation. Furthermore, each image has heavily cluttered backgrounds consisting of random day-to-day objects. Linemod-Occluded (Brachmann et al., 2014) is a subcategory of the Linemod dataset, where all objects in each image are occluded instead of just one, which creates a more challenging dataset as occlusion is added to the masks. The Linemod and Linemod-Occluded datasets are commonly used by other state-of-the-art methods for evaluation.

In this paper, we propose a method for the 6D pose estimation of textureless objects from a single monocular image. Our method is fast, robust, and requires only a CAD model of the object of interest for training.

## 3 Methodologies

In this section, we describe our proposed methodologies for 6D object pose estimation. We begin by defining the method’s main phases and then each phase in detail, including the offline template database generation and the online pose estimation approach.

### 3.1 Problem statement

**FIGURE 1:**
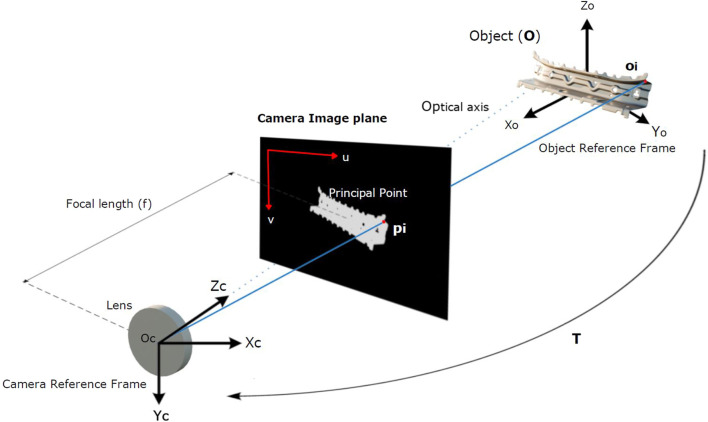
Pinhole camera model relevant to both the offline database generation and the online inference from a 2D camera. Masked images on the camera image plane are the input of our algorithm, which aims to estimate the object-to-camera pose T , regardless of the absolute system’s position on the world coordinate system.

In this work, we tackle the problem of estimating the 6D pose of industrial, textureless objects while keeping the complexity of the input hardware as low as possible, i.e., we rely on a single industrial monocular RGB camera, as shown in Figure 1. We also reduce the time complexity of the inference phase to allow deployment on embedded computing devices in the context of automotive production. In the following problem statement, we consider two algorithmic stages:

• **Offline phase**: A database comprising multiple viewpoints of an object of interest is generated from an available geometric CAD model.
• **Online phase**: Two-dimensional images are acquired from a monocular 2D camera, and with this input, a segmentation algorithm generates non-perfect segmentation masks of the object. The resulting binary masks are the input of our 6D pose estimation algorithm.

Figure 1 shows the basic elements considered in this problem statement for both the **online** and **offline** stages of our algorithm. Note that even though the world coordinate system is the general reference, it is not relevant to find a 6DOF translation matrix between an object and the camera. Thus, in this work, we focus on finding the object-to-camera transformation function without considering where the system is placed with respect to the world coordinate system. The input of our algorithm is on the two-dimensional image plane. Let O be a discrete set of visible surface points belonging to an object in the Euclidean three-dimensional space $R^{3}$ , as defined in Equation 1.

$$O=\left\{o_{i}\in R^{3}\;\text{with}\;i=1,\ldots ,N_{o}\right\}$$ (1)

Our goal is to find an object-to-camera *transformation function* in the special Euclidean group (Blanco-Claraco, 2022), $\;T=\left[R\;|\;t\right]\in SE\left(3\right):\;R^{3}\to R^{3}$ , that maps arbitrary points from the object to the camera reference frame according to Equation 2.

$$c_{i}=\left[R\;|\;t\right]\;o_{i}=T\;o_{i}$$ (2)

**FIGURE 2:**
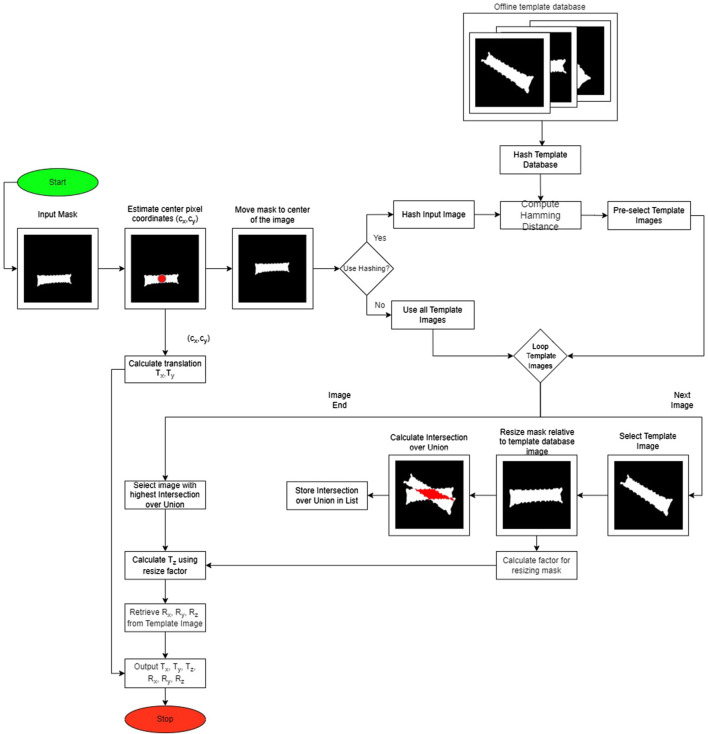
Full pipeline of our algorithm. The input is a binary mask of the object, which we match against rendered masks contained in an offline database. If hashing is used, then only a preselected number of images are matched instead of the whole database.

To solve the given problem statement, we used a template-matching method suggested by Druskinis et al. (2023). As shown in Figure 2, the main idea of template matching is to rely on a template database containing the object’s masks seen from different viewpoints. Then, during the online estimation phase, we aim to find the best match between the segmented object, i.e., input mask, and the masks in the database. The method is split into an offline and online stage, which is explained in the following sections.

### 3.2 Object template generation

Let us consider a pinhole camera (Harltey and Zisserman, 2006) to model how 3D points project to a 2D image plane (see Figure 1). During the offline phase, for each point $o_{i}$ belonging to the CAD model of the object of interest, we project the point to the image plane according to Equation 3.

$$p_{i}=K\;\left[R\;|\;t\right]\;o_{i}=K\;T\;o_{i}$$ (3)

Where $p_{i}=\left(u_{i},v_{i}\right)$ denotes the projected point in pixel coordinates, i.e., in the 2D camera image plane, and $K\in R^{3\times 3}$ represents the intrinsic camera matrix (Harltey and Zisserman, 2006). Equation 4 denotes the set of all projected points in the image plane, in pixel coordinates.

$$P=\left\{p_{i}\in N^{2}\;\text{with}\;i=1,\ldots ,N_{o}\right\}$$ (4)

The corresponding binary image mask is denoted in Equation 5, where |*B*| represents the resolution (i.e., the number of pixels) of the binary image mask.

$$B=\left\{\begin{array}{cc} 1 & \forall_{p_{i}\in P}\\ 0 & \text{other}\;\text{wise}, \end{array}\right\}$$ (5)

### 3.3 Offline database generation

In the offline phase, our method creates a binary image database of the object of interest seen from various viewpoints. We utilize a geometric model (CAD) of the object of interest and a rendering engine to generate multiple templates, i.e., binary image masks, corresponding to different object orientations.

More specifically, the binary image templates are obtained by discretizing the orientation space of the object, using an Euler angle representation, with $\theta_{x}\in \left[0,\pi \right]$ , $\theta_{y}\in \left[0,2\pi \right]$ , and $\theta_{z}\in \left[0,2\pi \right]$ denoting roll, pitch, and yaw, respectively, and projecting the rotated object geometric model in the image plane, as described in Section 3.2. The quantization steps are expressed in Equation 6.

$$\theta_{x}^{\text{step}}=\frac{\pi }{N_{\theta_{x}}},\theta_{y}^{\text{step}}=\frac{2\pi }{N_{\theta_{y}}},\theta_{z}^{\text{step}}=\frac{2\pi }{N_{\theta_{z}}}$$ (6)

$N_{\theta_{x}}$ , $N_{\theta_{y}}$ , and $N_{\theta_{z}}$ are user-defined discretization parameters.

The template database, D , is thus a set of binary masks representing the silhouette of the object seen from different orientations and is defined according to Equation 7.

$$D=\left\{B_{i,j,z}\;\text{with}\;i=1,\ldots ,N_{\theta_{x}},\;j=1,\ldots ,N_{\theta_{y}},\;z=1,\ldots ,N_{\theta_{z}}\right\}$$ (7)

**FIGURE 3:**
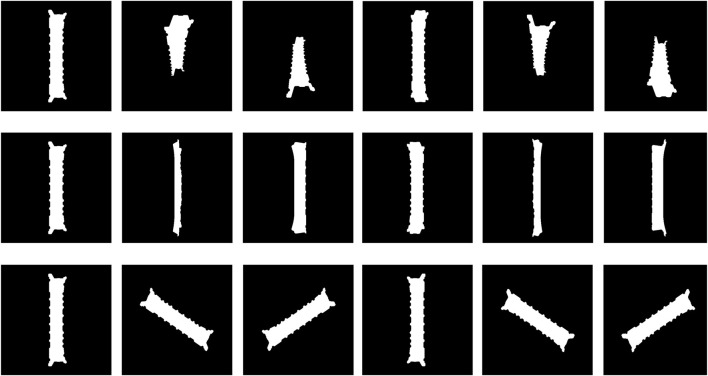
Images generated for the template database. Each row shows the object’s rotation on each axis: roll (X), pitch (Y), and yaw (Z). The object was rendered at 0.65 m from the camera to ensure that the entire object was in frame at every chosen orientation.

This comprises a total of $N_{\theta }=N_{\theta_{x}}\times N_{\theta_{y}}\times N_{\theta_{z}}$ orientation bins (as shown in Figure 3). The main difference in this approach compared to that used by Druskinis et al. (2023) is that the viewpoints of the object are not randomly sampled but instead created based on a deterministically defined 3D grid. Furthermore, we do not store edges of the object’s contours in the dataset but binary segmentation masks.

### 3.4 Template-based 6D pose estimation

In the online pose estimation stage, our algorithm receives an input binary image, given by a segmentation algorithm, and finds the best match with the database created in the offline stage (see Figure 2).

As shown in Figure 2, the first step of the online stage is to estimate the center pixel coordinates of the mask. Then, the center of the mask is aligned with the image’s center for subsequent computation of the 3D translation of the object. To do so, the centered binary mask is matched with the offline template database. For efficient computation, the hash number of the input images is computed. This input hash is compared against the mask database using a cost-effective Hamming distance. The templates with the lowest Hamming distance to the input image are preselected for further processing. Before the matching step begins, the input image is resized to have the same number of pixels as the template image. After resizing, the intersection over union cost function is used. The template that maximizes this cost function is selected as the best-matching mask. Then, the predicted rotation is retrieved from the database information on this winner template. The translation result is calculated from converting the previously estimated center pixel from the pixel space to the 3D world by using the camera’s intrinsic function. Lastly, the depth is calculated from the resize factor computed before IoU matching. The final output is the 6D pose of the object relative to the camera’s coordinate system, as shown in Figure 2.

#### 3.4.1 Perceptual hashing of binary images

Perceptual hashing (Mıhçak and Venkatesan, 2001) allows us to compare two images based on the underlying scene content instead of a purely numeric comparison of pixel values. It enables image comparison even in the presence of pixel modifications such as compression, color shifts, cropping, and rotation. This higher-level assessment of image contents has enabled perceptual hashing to be widely used in fields where comparing a massive amount of data is required in an efficient and robust manner, such as multimedia content grasping and identification (Farid, 2021).

In general terms, an ideal hashing algorithm should be characterized by a set of properties. The most important of them for our purposes can be described as follows: it must be **distinctive**, which implies that it must provide a distinguishable result for inputs with different content; it should also be **resilient** to pixel value modifications that do not fundamentally alter the underlying content; a perceptual hashing algorithm must also offer a **deterministic** output for invariant content; and finally, it must be **efficient** in terms of the cost of extracting and comparing hashes (Farid, 2021).

**FIGURE 4:**
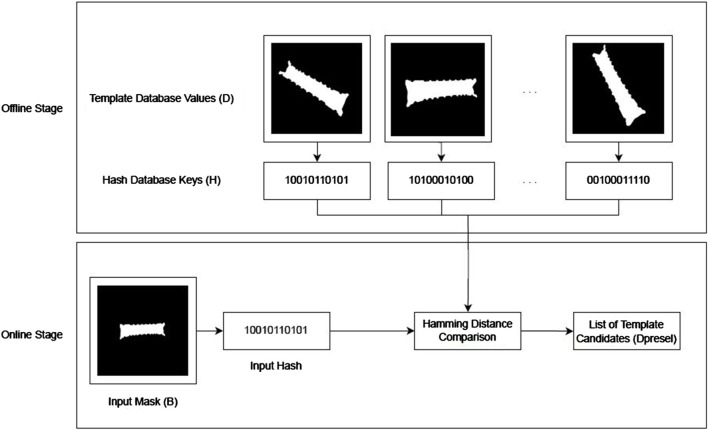
Representation of the offline and online steps involved in the hash-based preselection mechanism to create a subset $D_{\mathrm{presel}}\subseteq D$ of template candidates as the first step in our pose estimation algorithm.

Given the abovementioned **distinct**, **resilient**, **deterministic**, and **efficient** properties of perceptual hashing, we used this method to optimize the image search in our large template database in the field of pose estimation. To do so, we extracted hashes of all templates present in the dataset and saved a hash table in the offline stage, as shown in Figure 4. Then, at inference time, we computed once the hashing number of the input mask and used the database hashing table to search for hashes with the lowest Hamming distance with respect to the input mask. Templates with the smallest Hamming distance were then retrieved from the database and kept as candidates for the final IoU-based cost computation. This hash-based template preselection optimizes the brute-force search of the previous work (Druskinis et al., 2023).

There are multiple available implementations of perceptual hashing algorithms, such as average hash (aHash), perceptive hash (pHash), and difference hash (dHash) (Fei et al., 2017). We implement a modified version of aHash as its simplicity offers a reduced computation time compared to other methods while also offering good results in terms of the abovementioned properties (Fei et al., 2017).

**FIGURE 5:**
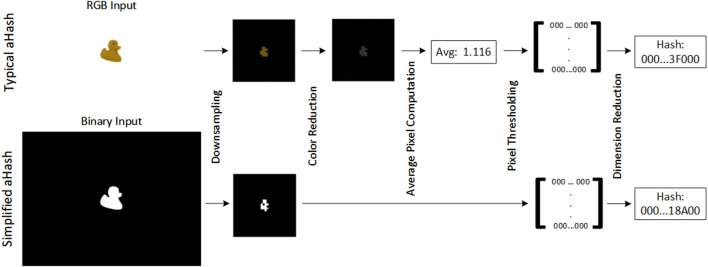
Step-by-step representation of the typical implementation of average hash (top) and our optimized version for binary inputs (bottom). We demonstrate the algorithm with object 9 of the Linemod (Hinterstoisser et al., 2013) dataset.

Average hashing mainly extracts features from low-frequency information in the image. Typically, it has four computation steps (Fei et al., 2017), as shown in the top of Figure 5, i.e., image downsampling, color reduction, average pixel computation, and pixel thresholding. However, considering a binary input, such as the binary mask B as defined by Equation 5, we can optimize the traditional aHash implementation for our specific inputs in two ways, as shown in the bottom of Figure 5. First, as the input B is already a single-channel binary mask, we do not need to reduce the input’s color dimensionality from RGB to grayscale. Second, if the nearest neighbor method is used as the interpolation method in the first step of the algorithm, the resulting lower-resolution mask keeps its binarized pixel values, which allow us to skip the subsequent pixel binarization step via a thresholding operation. Thus, our implementation of aHash can be summarized as follows:

1. Given an input binary mask B , its resolution is reduced by several orders of magnitude by using a two-dimensional interpolation method, such as nearest-neighbor interpolation. The resulting downscaled and binary image is further denoted as G .
2. The dimensionality of the resulting binary image G is reduced into a single-dimension binary code. The resulting binary number h represents the perceptual hash of the input image. The computed hash number h should have bits of several orders of magnitude lower than the original binary image mask B , i.e., |h|≪|B| .

As shown in the offline stage of Figure 4, we compute the above steps over all templates in our template database D to create a perceptual hash set, given by Equation 8, where *aHash* is the algorithm defined in the two abovementioned steps.

$$H=\left\{h_{j}=aHash\left(D_{j}\right)\;\text{with}\;j=1,\ldots ,|D|\right\}$$ (8)

Furthermore, as shown in Figure 4, during the online stage, we select a subset of template candidates from the database D by following the next two steps.

1. For a given input perceptual hash, $h_{\text{input}}$ , we compute the Hamming distance $\delta_{{\text{Hamming}}_{j}}$ (Hamming, 1986) between all elements in our perceptual hash set, H , by counting the number of binary elements that are different in each hash array according to Equation 9.

$$\Delta_{\text{Hamming}}=\left\{\delta_{{\text{Hamming}}_{j}}=|\left\{i\in \left\{1,\ldots ,n\right\}\;|\;h_{j_{i}}\neq h_{{\text{input}}_{i}}\right\}|\;\text{with}\;j=1,\ldots ,|H|\right\}$$ (9)

Where n represents the length of the binary perceptual hash arrays.

2. The elements in the database D are then sorted according to their Hamming distance values in $\Delta_{\text{Hamming}}$ . Consequently, the top mask templates, i.e., $|D_{\text{presel}}|=\alpha |D|\;$ are selected as the best candidates for further template matching. The variable α∈[0,1] represents the ratio of preselected elements. It can be defined by the user to adjust our search algorithm for maximum speed or higher accuracy, as demonstrated in Section 4.5.1, specifically in Figure 11. The preselection set is hence defined in Equation 10.

$$D_{\text{presel}}=\left\{D_{j}\;\text{with}\;j=1,\ldots ,\alpha |D|\right\}$$ (10)

Comparisons between one-dimensional hashes allow for a fast preselection of a fraction of template candidates $D_{\mathrm{presel}}$ from D , where $\left|D_{\mathrm{presel}}|\ll |D\right|$ , at the cost of minor accuracy losses (see Section 4.5.1). In other words, the main benefit of using perceptual hashing, as an early step in our pose estimation algorithm, is to provide an efficient way to preselect a subset of likely orientations (i.e., template candidates $D_{\mathrm{presel}}$ ) from the database D , using a single-dimension hash code and Hamming distances for similarity measure.

In the next section, we introduce a suitable similarity metric applied after the preselection step to select the winner from $D_{\mathrm{presel}}$ , with a metric able to differentiate subtler mask differences than the coarse hashing-based preselection is able to distinguish.

#### 3.4.2 Similarity cost function

As introduced in Equation 2, given a binary image representing the observed object mask, $\hat{B}$ , our goal is to estimate a SE(3) transformation $\hat{T}$ seen in Equation 11, that minimizes the error between $\hat{B}$ and a template transformation $B_{i}\subseteq D$ by using a suitable cost function C (Talak et al., 2023; Yang et al., 2023).

$$\begin{array}{c} \hat{T}=\underset{i}{argmin}\left(C\left(\;B_{i},\;\hat{B}\right)\right)\;\forall_{B_{i}\subseteq D} \end{array}$$ (11)

As addressed in Section 3.2, our pose estimation algorithm relies on a simple 2D input to tackle the 6DOF pose estimation problem. Additionally, we aim to keep the time complexity of our approach as low as possible to guarantee real-time inference on embedded computing devices. Thus, a suitable lightweight cost function that accepts such a 2D input and is agnostic to the object’s texture must be developed.

In our previous work (Druskinis et al., 2023), we used the Chamfer distance metric (Choi and Christensen, 2012) as a cost function for template matching. This function takes as input two binary images B and $\hat{B}$ and computes their similarity according to Equation 12.

$$\begin{array}{c} d_{\mathrm{chamfer}}\left(B,\hat{B}\right)=\frac{1}{\left|B||\hat{B}\right|}\underset{p_{i}\in B}{\sum }\underset{p_{j}\in \hat{B}}{\min}\;d_{E}^{2}\left(p_{i},p_{j}\right)=\frac{1}{\left|B||\hat{B}\right|}\underset{p_{i}\in B}{\sum }\underset{p_{j}\in \hat{B}}{\min}\;\|p_{i}-p_{j}{\|}_{2}^{2} \end{array}$$ (12)

Where $d_{E}\left(.\right)$ denotes the Euclidean distance between two points. The Chamfer distance has quadratic computation time complexity (Bakshi et al., 2024), i.e., $O\left(B\times \hat{B}\right)$ , due to the nested combination of sum and minimum search within the function across the elements contained in B and $\hat{B}$ .

In this work, we avoid expensive computations involved in edge extraction and rely on a simple intersection over union as our cost function, defined according to Equation 13.

$$\begin{array}{c} IoU\left(B,\hat{B}\right)=\frac{B\cap \hat{B}}{B\cup \hat{B}} \end{array}$$ (13)

Therefore, the pose estimation problem is reduced to estimating which binary image in the database best overlaps the observed one, as seen in Equation 14.

$$\begin{array}{c} \hat{T}=\underset{i}{argmax}\left(\frac{B\cap \hat{B_{i}}}{B\cup \hat{B_{i}}}\right)\;\forall_{\hat{B_{i}}\subseteq D} \end{array}$$ (14)

## 4 Results

In order to evaluate the proposed pose estimation methods, we generated synthetic object views, corresponding to different relative camera–object poses, using a realistic rendering engine, to have groundtruth pose, noise and occlusion, which are difficult to acquire in real-life experimental scenarios.

As the segmentation algorithm is beyond the scope of this work, we directly rendered synthetic masks in the image plane. In all our experiments, we used a realistic open-source rendering engine, BlenderProc (Denninger et al., 2023b), to generate binary image masks representing different views of the object of interest.

In the rest of this section, we evaluate the robustness of our algorithms in the presence of synthetically generated occlusions and noise, as well as the computational gains of the proposed perceptual hashing for binary images. Finally, we measure the processing time of the proposed methods on different computing devices.

### 4.1 Evaluation metrics

In this work, we use two metrics for evaluating the translation and rotation error of inferred poses of a given test set $D_{\mathrm{test}}$ compared to a ground truth, as described by Hodan et al. (2016). Following the definition of a transformation function $\;T=\left[\;R\;|\;t\;\right]\in SE\left(3\right)$ defined in Equation 2, we denote a groundtruth translation matrix as $\bar{t}$ and an estimated translation matrix as $\hat{t}$ , both in $R^{3}$ . The Euclidean distance is used to calculate the translation error in meters (m) between the groundtruth and predicted translation vectors as defined in Equation 15.

$$e_{\text{trans}}\left(\hat{t},\bar{t}\right)=\underset{x\in D_{\mathrm{test}}}{avg}\|\hat{t}-\bar{t}{\|}_{2}$$ (15)

Similarly, we define a groundtruth rotation matrix, $\bar{R}$ and an estimated rotation matrix, $\hat{R}$ , and compute the average rotation error as per Equation 16, across a test set $D_{\mathrm{test}}$ given by the angle in degrees.

$$e_{\text{rot}}\left(\hat{R},\bar{R}\right)=\underset{x\in D_{\mathrm{test}}}{avg}arccos\left(\frac{Tr\left({\hat{R}\bar{R}}^{-1}\right)-1}{2}\right)$$ (16)

For simplicity, our implementation represents the Euler angles of $\bar{R}$ and $\hat{R}$ as quaternions and calculates Equation 16 as the error between two quaternions by computing the Hamilton product according to Equation 17.

$$q\Delta =q_{2}q_{1}^{-1}$$ (17)

### 4.2 Experimental setup

As shown in Figures 2, 4, our algorithm consists of an offline database generation and an online matching stage. For both, we define a set of parameters that are kept invariant during the experimental phase, unless otherwise explicitly stated in a particular experimental scenario. In particular, for the template database D generation, we quantize the orientations in steps of 10° as seen in Equation 18.

$$\theta_{x}^{\text{step}}=\theta_{y}^{\text{step}}=\theta_{z}^{\text{step}}=10{}^{\circ}$$ (18)

This is achieved by setting the rotation discretization parameters to the values seen in Equation 19.

$$N_{\theta_{x}}=18\;\text{and}\;N_{\theta_{y}}=N_{\theta_{z}}=36,$$ (19)

yielding a total of $|D|=|H|=N_{\theta_{x}}\times N_{\theta_{y}}\times N_{\theta_{z}}=23328$ elements in the database.

A base binary mask testing set was further created to not contain the same images as the template database. Instead, these were generated with a 1° offset from the template database to avoid trivial matches.

Thus, the testing set was generated with the following rotations: $\theta_{x}\in$ [−21°,19°] , $\theta_{y}\in$ [−21°,19°] , and $\theta_{z}\in$ [−1°,359°] with a rotation step of 12 .

Furthermore, to test the translation accuracy of our pose estimation method, the object was further translated in the images with the translations X∈[−0.25m,0.25m],Y∈[−0.25m,0.25m],Z∈[0.65m,2m] while discarding views in which the object was only partially visible, i.e., not fully within the field of view of the simulated camera.

To assess the robustness of our method, the binary images in the base test set were further corrupted with different levels of artificially generated occlusions and noise.

In this section, we evaluate the accuracy of our pose estimation approach in the presence of occlusion and noise by artificially generating occlusions and noise over the input testing masks.

### 4.3 Robustness against occlusion

**FIGURE 6:**
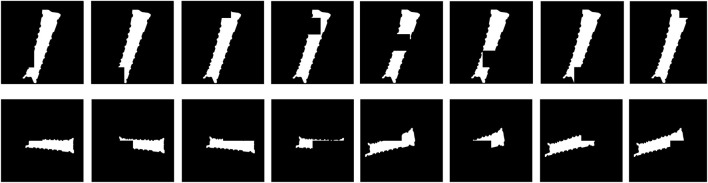
Occlusion of a mask using the sliding box approach. The first row contains one mask with an occlusion box with 10% of the size of the image. The second row contains two different masks with an occlusion box size of 25% of the image.

In order to test the robustness of the pose estimation method to different occlusion levels, each image in the base testing set was occluded using a variable-size black sliding window. The occlusion levels ranged from 0% to 100% depending on the amount of occlusion (i.e., overlap) between the sliding window and the original binary mask (see Figure 6).

**FIGURE 7:**
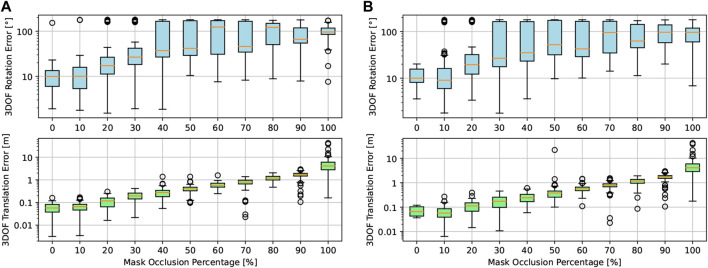
Logarithmic representation of absolute rotation and translation errors at occlusion levels ranging from 0% to 100% with and without dataset preselection based on perceptual hashing. **(A)** Absolute rotation and translation errors vs occlusion levels only using IoU as the matching method. **(B)** Absolute rotation and translation errors vs occlusion levels using image hashing as preselection and IoU as the matching method.

In Figure 7, we evaluate the accuracy behavior of our approach in terms of absolute rotation and translation errors in the presence of the abovementioned occlusion levels. On the left, only IoU is used as the cost function, whereas on the right, we first filter template candidates with perceptual hashing, only allowing 10% of the original amount of images in the dataset to be evaluated with IoU. The results for every occlusion level are plotted as distributions in the logarithmic scale, while outliers are marked as points outside it.

Rotation and translation errors are low (around 10° ) in the presence of low levels of occlusion (i.e., the first two sample bins); however, their average exhibits a linear increase afterward proportionally to the occlusion level. Additionally, the similarity between the results in the left and right plots shows that the accuracy in the presence of occlusion is not degraded even if we use our preselection perceptual hash to filter out 90% of the binary image masks in the database, before the refined IoU-based matching-based pose estimation takes place.

### 4.4 Robustness against noise

**FIGURE 8:**
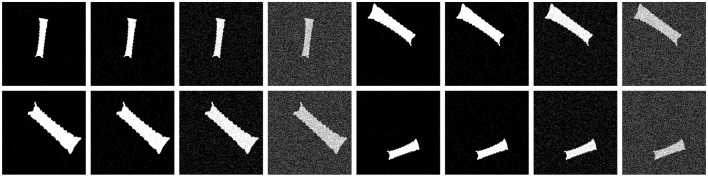
Gaussian noise added to four example masks (two masks in each row), with a signal-to-noise ratio (SNR) varying between 20 dB, 15 dB, 5 dB, and −5 dB (from left to right).

Similar to the approach followed in Section 4.3, we characterize the behavior of our algorithm in the presence of multiple noise levels. Given a binary image mask B , as defined in Equation 5, we apply binary noise with a signal-to-noise ratio (SNR) defined by Equation 20. Note that, as shown in Figure 8, a higher SNR means a cleaner mask, whereas a negative value means that the number of mask pixels is smaller than the number of noisy pixels.

We applied the binary salt and pepper method suggested by Zhou and Gordon (1991) for random noise generation (see Figure 8 for generated masks), where the SNR is calculated using Equation 20.

$$SNR=10\cdot log_{10}\left(\frac{\sigma_{s}^{2}}{\sigma_{n}^{2}}\right)$$ (20)

Here, $\sigma_{s}^{2}$ and $\sigma_{n}^{2}$ are the variance of the signal and the variance of the noise, respectively, as seen in Equations 21, 22. $N_{0}$ denotes the number of pixels belonging to the object, and $N_{n}$ denotes the number of pixels for the noise, i.e., the number of pixels that have been altered. N is the total number of pixels in the image.

$$\sigma_{s}^{2}=\frac{N_{0}}{N}\left(1-\frac{N_{0}}{N}\right)$$ (21)

$$\sigma_{n}^{2}=\frac{N_{n}}{N}\left(1-\frac{N_{n}}{N}\right)$$ (22)

**FIGURE 9:**
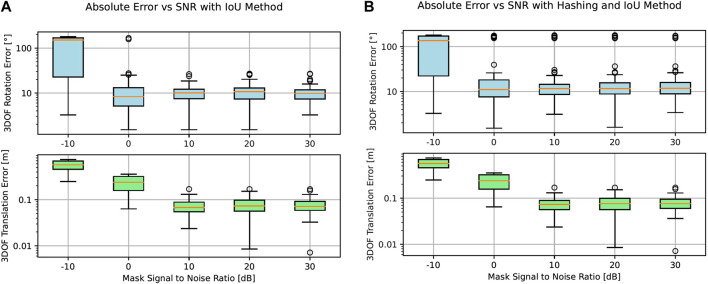
Absolute rotation and translation errors at signal-to-noise levels ranging from −10 dB to 30 dB. In **(A),** there is no hashing preselection, whereas in **(B),** a hashing preselection of 10% of the original database is taken. **(A)** Absolute rotation and translation errors vs signal-to-noise levels only using IoU as the matching method. **(B)** Absolute rotation and translation errors vs. signal-to-noise levels using image hashing as preselection and IoU as the matching method.

Figure 9 shows the accuracy of our pose estimation algorithm in the presence of binary noise with SNR ∈[−10,0,10,20,30] . On the left side, we observe that both the rotation and translation errors remain stable from 30 to 10 dB, and at a noise level of 0 dB, only the translation error increases.

On the right side of Figure 9, we observe a similar behavior in the presence of noise, even if in this case, we filter 90% of the original database with the perceptual hashing method discussed in Section 3.4.1 before starting to evaluate the remaining 10% of image candidates with IoU.

With an extreme SNR worse than 0 dB, where the amount of noise predominates over the information on the image, our algorithm reaches its limits and does not provide satisfactory results.

It is noteworthy that these tests were made with a template database with a quantization step of 10° in the Euler rotation space, as described in Equation 6, and a hashing preselection of only 10% for fast computation. One should expect lower errors if lower rotation quantization steps are set at the cost of higher inference time.

### 4.5 Image hashing analysis

As stated in Section 3.4.1, we investigate the usage of perceptual hashing to avoid brute-force search of large template databases needed for a 6DOF pose estimation algorithm introduced by Druskinis et al. (2023). To do so, we propose a template preselection phase, as shown in Figure 2, where, based on perceptual hashing features, the preselection stage identifies template candidates with similar rotations.

**FIGURE 10:**
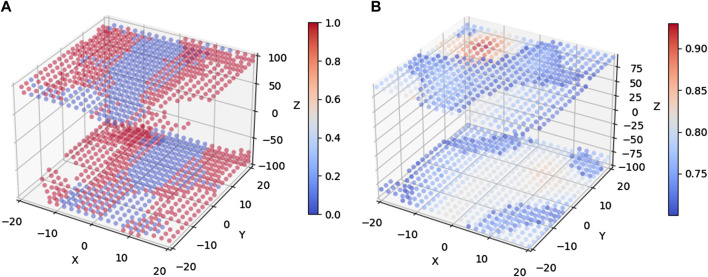
Cost functions based on Hamming distances of perceptual hashes and IoU over the three-dimensional rotation space of the template database for an input image with a groundtruth rotation given by $\left(\theta_{x},\theta_{y},\theta_{z}\right)=\left(-10{}^{\circ},10{}^{\circ},85{}^{\circ}\right)$ . **(A)** Hamming distance of image hashing over the template dataset with a distance smaller than 2. Note the limited resolution leading to many templates having the same score. **(B)** IoU cost of template candidates from the hashing stage with a score greater than 0.7. Note a higher IoU score resolution than that in **(A)**.

Depending on the rotation quantization steps and the range of allowed viewpoints in the rotation space, the database can potentially contain thousands of templates to be compared. For instance, Figure 10 shows a 3D visualization of the three-dimensional rotation space in Euler angles of a dataset with $\theta_{z}\in \left[-100{}^{\circ},100{}^{\circ}\right],\;\theta_{y}\in \left[-20{}^{\circ},20{}^{\circ}\right]\;\text{and}\;\theta_{x}\in \left[-20{}^{\circ},20{}^{\circ}\right]$ degrees in a spherical grid of viewpoints with 0.65 m radius and a rotation quantization step of 2°.

In this $R^{3}$ space, we take a sample test input located at rotations $\left(\theta_{x},\theta_{y},\theta_{z}\right)=\left(-10{}^{\circ},10{}^{\circ},85{}^{\circ}\right)$ and visualize its similarity to the multiple templates in the database by using perceptual hashing (Figure 10A) and IoU (Figure 10B) as cost functions.

More specifically, in Figure 10A, we apply perceptual hashing and calculate the distance of resulting binary hash numbers by using Hamming distance; then, only templates with a Hamming distance <2 (template candidates) are plotted to reduce cluttering. On the other hand, in Figure 10B, we re-evaluate the resulting template candidates from the previous preselection stage with the IoU cost function and plot the location of the resulting templates with an IoU greater than 0.7.

As shown in Figure 10A, the Hamming distance metric compares two binary numbers and outputs an integer result, providing coarse resolution for images with similar perceptual hashing numbers. This explains why many templates share the slowest distance of 0 with respect to the input mask, which are plotted as blue points in the graph. This shows that even though perceptual hashing offers a way to filter out more than 90% of the templates in the dataset for this specific example, the discrete nature of its result makes it hard to use it to directly select a single winner template with the smallest distance. Thus, a cost function, such as IoU, which allows evaluation refinement of template candidates from the preselection step, is required. As shown in Figure 10B, the red hotspot offers a clearer winner template close to the $\left(\theta_{x},\theta_{y},\theta_{z}\right)=\left(-10{}^{\circ},10{}^{\circ},85{}^{\circ}\right)$ ground truth.

#### 4.5.1 Computational complexity and pose accuracy

**FIGURE 11:**
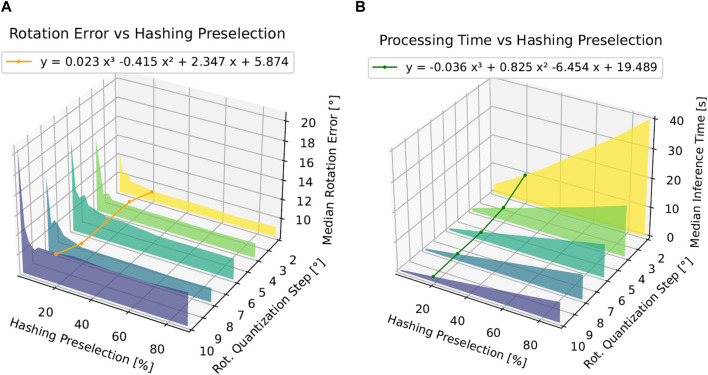
Inference time and median rotation error as a function of the hashing preselection ratio α∈[0.1,0.9] and database quantization step $\theta_{x}^{\text{step}}=\theta_{y}^{\text{step}}=\theta_{z}^{\text{step}}\in \left[2{}^{\circ},10{}^{\circ}\right]$ . **(A)** Median inference time (s) as a function of the hashing preselection percentage and the dataset quantization step. Furthermore, we draw a polynomial fitted line for hashing preselection at 20%. **(B)** Median rotation error as a function of the hashing preselection percentage and the dataset quantization step. Furthermore, we draw a polynomial fitted line for hashing preselection at 20%.

In this section, we evaluate different levels of computational complexity (inference time) and its relationship with the resulting pose accuracy. As shown in Figure 11, we base our benchmarking on the discrete variation in two variables, i.e., the dataset quantization $\theta_{x}^{\text{step}}=\theta_{y}^{\text{step}}=\theta_{z}^{\text{step}}\in \left[2{}^{\circ},10{}^{\circ}\right]$ , as defined in Equation 6, and the ratio of hashing preselection α∈[0.1,0.9] .

We observe on the left in Figure 11 that a lower hashing preselection ratio allows us to meet the condition $\left|D_{\mathrm{presel}}|\ll |D\right|$ , as described in Section 3.4.1, saving computation time across all database quantization levels. For instance, for a database created with 2° quantization step, if we compare the inference time between α=0.9 and α=0.2 , we observe a four-fold inference time reduction.

On the right side of Figure 11, we observe no significant accuracy improvement if α>0.2 . Based on this, we observe that we are able to reduce four times the inference time with virtually no loss in accuracy by using our perceptual hashing preselection step.

#### 4.5.2 Inference time on different computing devices

Table 1 shows a comparison of the computation time of our algorithm in multiple computing devices, comprising a workstation, an industrial NUC, and two embedded computing devices. It is noteworthy that our implementation runs completely on the CPU, which means that the GPU of the shown devices does not play a role in the time benchmarking. However, as our CPU-based implementation is optimized to run in parallel threads, the amount of physical and virtual cores plays a key role in the shown timings.

**TABLE 1:** Results of our method on different computing devices. Each device has the CPU architecture, cores, type, and frequency noted.

	Workstation	NVIDIA AGX Orin	Intel NUC	Raspberry Pi 4
Average inference time [s]	0.210	0.297	0.520	1.52
CPU architecture	x86_64	aarch64	x86_64	aarch64
CPU cores	20	12	4	4
CPU type	Intel i9-9820X	Cortex-A78AE	Intel i5-7300U	Cortex-A72
CPU frequency [GHz]	3.3	2.2	2.6	1.5

In our implementation, we first load the database D in the RAM of the shown devices to avoid slow accesses to the persistent memory. Thus, the template dataset used in these tests has been designed to fit in the available RAM of all devices, comprising, in total, 2,916 images. This also corresponds to a viewpoint range of $\theta_{x}\in$ [0°,90°] , $\theta_{y}\in$ [0°,90°] , and $\theta_{z}\in$ [0°,360°] , which is in line with datasets based on viewpoints on the upper hemisphere, such as Linemod (Hinterstoisser et al., 2013). Additionally, to be consistent with the accuracy results shown in Figure 7 and Figure 9, we use a quantization step of 10°.

The frame rate of our pose estimation algorithm is around 4,7 frames per second (FPS) on a modern workstation without GPU support and at 3,3 FPS on a modern embedded device. To compare our method against that suggested by Druskinis et al. (2023), we measured the inference time on the same workstation under the same conditions and measured an inference time of 47.8 s per image, which translates to 0.02 FPS. Thus, our novel implementation exhibits a processing time improvement of two orders of magnitude while delivering similar average rotation and translation accuracy.

To further emphasize the importance of fast pose estimation, consider our specific use case of the automotive production in Mercedes-Benz AG automated factories in Sindelfingen, where, for instance, the pose of a body-in-white part being transported by an automated guided vehicle (AGV) must be computed at a quality gate where the AGV stops for 4 s. Under such conditions, a total processing time of less than 4 s is enough to avoid delaying the advance of the AGV to a subsequent assembly line. Such a practical processing time requirement could be met by our algorithm even if deployed on a low-cost Raspberry Pi4.

### 4.6 Comparison against state-of-the-art methods

Our method has been compared against leading pose estimation algorithms to quantify the advantages and disadvantages between them. The BOP Challenge website offers results from various 6D pose estimation models that have been tested on popular 6D datasets. We tested our method on the Linemod-Occluded dataset, which is a large dataset containing 15 textureless household objects with discriminative color, shape, and size. The BOP challenge uses different evaluation metrics to assess pose accuracy from those used in our previous experiments; thus, we used their metric to directly compare our method with others. The error of an estimated pose with respect to the groundtruth pose is calculated using the following pose–error functions: Visible Surface Discrepancy (VSD), Maximum Symmetry-Aware Surface Distance (MSSD), and Maximum Symmetry-Aware Projection Distance (MSPD). VSD, defined in Equation 23, treats indistinguishable poses as equivalent by considering only the visible object part.

$$e_{\mathrm{V SD}}\left(\hat{D},\bar{D},\hat{V},\bar{V},\tau \right)=avg_{p\in \hat{V}\cup \bar{V}}\left\{\begin{array}{cc} 0,\; & \text{if }p\in \hat{V}∐\bar{V}\Lambda |\hat{D}\left(p\right)-\bar{D}\left(p\right)|<\tau \\ 1,\; & \text{otherwise}. \end{array}\right.$$ (23)

τ is the misalignment tolerance. The MSSD, defined in Equation 24, considers a set of pre-identified global object symmetries and measures the surface deviation in 3D.

$$e_{\mathrm{MSSD}}\left(\hat{P},\bar{P},S_{M},V_{M}\right)=min_{S\in S_{M}}max_{x\in V_{M}}\|\hat{P}x-\bar{P}Sx{\|}_{2}$$ (24)

The MSPD, defined in Equation 25, considers the object symmetries and measures perceivable deviation.

$$e_{\mathrm{MSPD}}\left(\hat{P},\bar{P},S_{M},V_{M}\right)=min_{S\in S_{M}}max_{x\in V_{M}}\|proj\left(\hat{P}x\right)-proj\left(\hat{P}Sx\right){\|}_{2}$$ (25)

An estimated pose is considered correct with respect to a pose–error function, e , if $e<\theta_{e}$ , where $\theta_{e}$ is a threshold of correctness and e∈VSD,MSSF,MSPD is a pose–error function. The fraction of object instances with a correct pose is referred to as recall. The average recall, AR , with respect to a pose–error function, e , is denoted as $AR_{e}$ and defined as the average of the recall rates calculated for multiple settings of the threshold $\theta_{e}$ . Finally, the accuracy for a dataset D , such as the Linemod-Occluded dataset (Brachmann et al., 2014), is measured by Equation 26, which is calculated over all the estimated poses of all objects from the Linemod-Occluded dataset. Our method was tested on the 15 objects from the Linemod-Occluded dataset (Brachmann et al., 2014) to benchmark our 6D pose estimation method against other reported state-of-the-art algorithms.

A template database of all 15 objects with Linemod-Occluded compatible viewpoints was generated with multiple quantization steps in {5°,10°,15°} . Our results were evaluated using the evaluation file from the BOP toolkit^1^. The results are given in Table 2, showcasing the average recall for each object depending on the quantization step used.

$$AR_{D}=\left(AR_{\mathrm{V SD}}+AR_{\mathrm{MSSD}}+AR_{\mathrm{MSPD}}\right)/3$$ (26)

**TABLE 2:** Results of the 15 objects from the Linemod-Occluded (Brachmann et al., 2014) dataset. The metric used is the average recall from the BOP metrics.

Object	1	2	3	4	5	6	7	8	9	10	11	12	13	14	15
AR	0.44	0.47	0.38	0.55	0.41	0.52	0.31	0.40	0.45	0.60	0.49	0.56	0.48	0.42	0.46

Table 3 shows the results comparing different 6D pose estimation methods on the Linemod-Occluded dataset.

**TABLE 3:** Results from various 6D pose estimation methods on the Linemod-Occluded dataset (Brachmann et al., 2014).

	MegaPose	CosyPose	Ours
$AR_{D}$	**0.65**	0.63	0.46
Inference time (s)	6.04	1.20	**0.21**

Bold values indicate the highest-performing method in each category.

**FIGURE 12:**
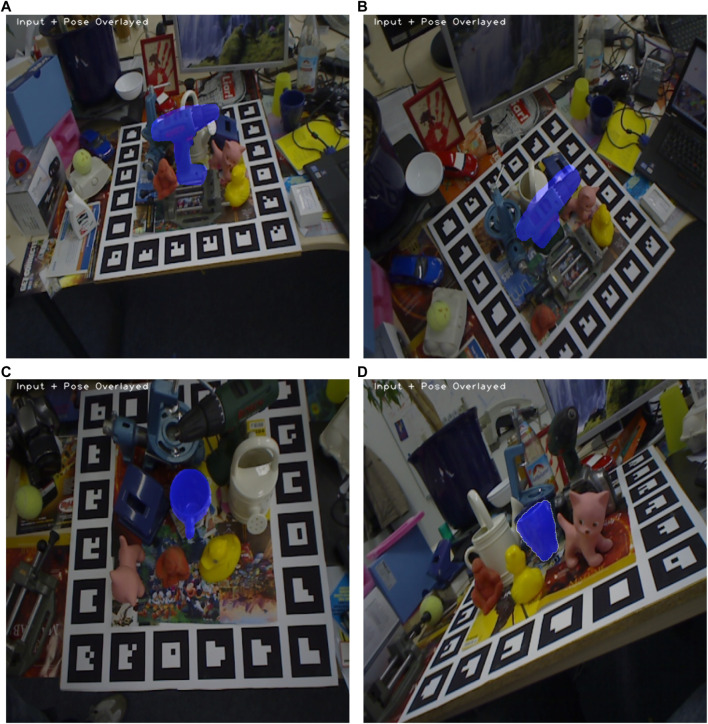
Predictions on the Linemod-Occluded dataset using our method. A blue mask indicates our prediction. **(A)** Good prediction on the drill (object 8) from the Linemod-Occluded dataset using our method. **(B)** Bad prediction on the drill (object 8) from the Linemod-Occluded dataset using our method. **(C)** Good prediction on the cup (object 7) from the Linemod-Occluded dataset using our method. **(D)** Good prediction on the cup (object 7) from the Linemod-Occluded dataset using our method.

Figure 12 shows some predictions using our method on the drill (object 8) and the cup (object 7) of the Linemod-Occluded dataset. Figure 12A shows a good prediction on the drill object, where the prediction mask is overlaid perfectly on the actual image. On the contrary, Figure 12B shows a bad prediction of the drill, where our method rotated the object 180° along the major axis, causing it to face the other way, resulting in a large rotation error. The cup object is also interesting as prediction is good when the handle is clearly visible, as shown in Figure 12C. However, our method fails on this object when the handle is not clearly visible and distinct in the mask, as shown in Figure 12D. This object is specifically challenging since there are many different angles where the cup can have the same mask due to the symmetry of the cup, which is also why the average recall on the cup is 0.29.

Furthermore, the processing time for one image for our method is 0.21 s, whereas MegaPose uses 6.04 s.

### 4.7 Discussion

As shown in Figure 11A, the computational inference time of our approach scales in an inversely proportional manner with the size of the template image database as the algorithm necessitates assessing all images within the database to identify the optimal match. Notably, as shown in the fitted polynomial curve in Figure 11A at a hashing preselection of 20%, the theoretical upper bound for the computing time corresponds to 19.489 s if the quantization step ≪10° , and it stands at 1.4 s for a quantization step of 10° . In line with this, as shown in Figure 11B, as the number of images increases, leading to a greater array of potential rotations, the mean rotation error decreases. For instance, as shown in the fitted polynomial curve of Figure 11B, at a hashing preselection percentage of 20%, the mean rotational discrepancy diminishes to a theoretical minimum of 5.87° with a quantization step size ≪10° of 2° and increases to a maximum of 10.84° with a quantization step size of 10° , accurately reflecting the precision gap for this quantization step. Moreover, the translation error remains constant, regardless of the step size and hashing preselection percentage as the method for computing translation is independent of the database, relying instead on determining the central pixel of the input mask and converting it to world coordinates. The average translation error of 0.1 m suggests improving potential in pinpointing the central pixel as it fails to correspond precisely with the center of the 3D object. However, considering the length of the object being approximately 74 cm, the relative translation error corresponds to 14% compared to its longitude.

Deviations observed in the test results highlight errors stemming from flipping as the algorithm struggles to differentiate between the correct template orientation and a template rotated by 180° due to symmetries in the object used for evaluation, which cause indistinguishable masks for different orientations.

The data shown in Figure 11 suggest that the integration of a hashing algorithm notably expedites processing time by preselecting a specific percentage of images from the database for comparison, rather than evaluating all images. This pre-selection step decreases the inference duration by two-fold with virtually no loss in accuracy for a preselection range [20%,100%] .

Additionally, as shown in Figure 7, our template matching methodology exhibits accurate performance in instances where the mask is significantly occluded. Notably, once 50% of the mask is obscured, the predictions become erratic due to insufficient data for accurate pose estimation. Similarly, as shown in Figure 9, our pose estimation approach exhibits robustness to decreasing SNRs, corresponding to increased image noise. In particular, rotation and translation errors stay approximately constant for up to 10 dB.

As shown in Table 3, our method achieves an average recall of 0.44 on the whole Linemod-Occluded (Brachmann et al., 2014) dataset using a quantization step of 10°, which translates into an inference time of approximately 0.2 s per image. CosyPose (Labbé et al., 2020) and MegaPose (Labbé et al., 2022) exhibit an average recall of 0.63 and 0.65, respectively, by leveraging complex learning-based algorithms. Even if we accelerate computation using an NVIDIA Quadro GV100 GPU, MegaPose requires 30 times more computation time compared to our method being executed exclusively on the CPU. As shown in Table 2 and Figure 12, object 10 has the highest average recall of 0.60 with our method, whereas object 7 achieves an average recall of 0.31. This major difference in the results can be because our template-based method relies on unique viewpoints of objects to correctly find the match, which is hard to achieve with object 7 due to a lack of distinct features that are always visible in the mask, making it harder to differentiate between different viewpoints. Although our results do not outperform other leading state-of-the-art methods, our approach can still have potential in cost-effective devices as we achieve a better processing time than the other methods, without the need for a GPU. Lastly, deep learning based-methods usually require training of their models and a large annotated dataset to train on, whereas our method only requires a CAD model of each object for the generation of a template database.

## 5 Conclusion and future work

In this work, we proposed a template-based approach for the 6D pose estimation of objects using monocular images.

Our new template-based matching algorithm utilizes binary image masks, representing the object silhouette, and perceptual hashing for fast inference. Our algorithm effectively filters out up to 80% of the template database, significantly enhancing the inference speed while maintaining a performance comparable to our previously proposed non-optimized version. Remarkably, it operates efficiently on diverse computing devices, even without GPU support, showcasing reduced runtime and high accuracy.

We evaluated our approach on a body-in-white metallic part relevant to automotive production, revealing superior trade-offs between accuracy and computation time compared to previous methods (Druskinis et al., 2023), as discussed in Section 4.5.2. Specifically, our algorithm achieves an average rotation error of approximately 10°, an average translation error of 14% of the object’s length, and an average processing time of 0.3 s per image on an NVIDIA AGX Orin device, i.e., two orders of magnitude faster than the previous algorithm, while maintaining accuracy. Furthermore, it demonstrates robustness against partial occlusion, retaining accuracy even with up to 10% occlusion levels, and against noisy image inputs with no loss of accuracy up to a 10-dB signal-to-noise ratio. Additionally, we compared our approach to other state-of-the-art-methods, CosyPose and MegaPose on the Linemod-Occluded dataset, and achieved an average recall of 0.46 compared to 0.63 and 0.65, respectively, with a processing time of 0.29 s compared to 1.2 s and 6.04 s, respectively.

Arguably, the main limitation to our lightweight 6D pose estimation method is its sole reliance on segmentation masks, making it impossible to distinguish different poses, resulting in equivalent silhouettes. In order to improve the accuracy in these cases, we propose refining our result using the object’s RGB information as a post-processing step, for instance, by using RGB-based key point descriptors.

## Data Availability

The raw data supporting the conclusion of this article will be made available by the authors, upon reasonable request.

## Footnotes

1
https://github.com/thodan/bop_toolkit
